# The Institutional Library

**Published:** 1915-04-03

**Authors:** 


### THE INSTITUTIONAL LIBRARY.

### Surgical Procedure.

Surgical Materials and Their Uses. By Alexander MacLennan, M.B., C.M. (Glasg.). (London: Edward Arnold. 1915. Pp. 247 + viii. Price 4s. 6d. net.)

Mr. MacLennan has not chosen the title of his book very happily. It suggests a manufacturer's catalogue rather than a book for medical students and practitioners. The book really belongs to the class which deal with surgical procedures other than actual operations. It is concerned with the art of bandaging, the application of splints and dressings, the uses of sutures and ligatures, the relative values of certain antiseptics, and the employment of a selected number of surgical instruments. But in each of these instances special attention is given to the physical qualities or methods of preparation of the materials concerned, and this feature certainly gives to the book an individuality which is not without its value. The text is very freely illustrated, and thus the book will help the student who desires to cultivate the classical style in bandaging—an ideal which Mr. MacLennan recognises has nowadays largely lost its attraction. In all the chapters are numerous practical directions likely to be of value to the student of surgery, and the book merits the attention of the class of reader which it seeks to serve.

### Mental Disorders.

Mental Medicine and Nursing. By Robert Howland Chase, A.M., M.D. (Philadelphia and London: J. B. Lippincott Co. Price 6s. net.)

Success in examinations by itself is not altogether a satisfactory criterion by which to judge the efficiency of the individual who has safely passed by the Scylla of the written portion and avoided the Charybdis of the *viva voce*! More especially is this the case if, in subjects where practical acquaintance is essential, the information obtained is merely so many words and phrases memorised, to be repeated later with all the accuracy of a phonographic record. It is being slowly realised that an accurate memory is only one part of the mental equipment, and that what is chiefly necessary is the power of selecting and utilising the information obtained. In the meantime, however, we have the educational methods we deserve; and in lecturing and teaching it is still expedient to sacrifice to the deities—or demons?—who preside over examinations in order to propitiate them. Even candidates who are efficiently trained and practically reliable have to perform the required lip-service, and for this purpose much book work is essential. Even in so specialised a department of nursing the range of knowledge has to be extensive; so extensive, indeed, that the lecturer and the learner have carefully to avoid the diffusion of their energies over too wide a field. This is especially to be guarded against in teaching—and in learning—psychology, which is now included in the curriculum of the mental nurse as a needful pre-requisite to the study of disorders of the mind. Dr. Chase's book deals with that subject in an extremely succinct and lucid way. It is so compressed, and withal so compendious, that one realises the amount of labour which must have been required thus to separate essentials from a vast, rather ill-defined, aggregation. This section of Dr. Chase's book should prove of particular value to those who have to instil the elements of psychology into classes the members of which have had little or no previous acquaintance even with the terminology of the subject; while to those in professional practice this terse and condensed account of normal mental processes should prove invaluable.

The same clarity is, fortunately, to be observed throughout. The symptoms of mental disorder are lucidly described; the forms of mental disease are briefly but adequately delineated. There are admirable chapters on treatment—"The Patient from the Physician's Viewpoint" and "The Patient from the Nursing Viewpoint," and there is a useful Appendix on poisons and antidotes. The book is excellently illustrated, and the portraits of cases should be useful in helping to recall to memory similar ones already noted in the course of clinical observation.

If a word of criticism may be hazarded, it is in regard to the list of definitions which Dr. Chase has included. Neologism, for instance, is not a "repetition of senseless expressions"; and, indeed, this definition is given by Dr. Chase to verbigeration. It may be pointed out that the repetition is not necessarily of "senseless" expressions. One or two more terms are rather loosely defined—*e.g.*, simulation, paramnesia. These are, however, small defects in a book which is worthy of a place in the library of anyone who is interested in the study or the teaching of the symptomatology and treatment of mental disorders.

### The S.P. Pocket Guide Series.

The Muscular System. By Harold Burrows, M.B., F.R.C.S. Illustrated. (The S.P. Pocket Guide Series.) (London: The Scientific Press, Ltd. 1915. Price 1s.)

Notes on the Nervous System. By Edwin Ash, M.D. (Lond.). Illustrated. (The S.P. Pocket Guide Series.) (London: The Scientific Press, Ltd. 1915. Price 1s.)

These two new additions to the series of handbooks issued by The Scientific Press are admirable examples of the compact utility which characterises these publications. There are now fifteen of these pocket guides on the market, and the success of the series is evidence of their appreciation by the nursing profession. Mr. Burrows has succeeded in giving in a brief yet very clear manner the most important features of the anatomy of the skeletal muscles, together with their functions and nerve supplies. A useful innovation is the classification of the muscles based upon function rather than upon anatomy; the individual muscles are described under the heading of the joint upon which they act. Due praise must be given for the clearness of the numerous illustrations, a feature which is all the more creditable in a book of this type, having regard to the smallness of the page.

"Notes on the Nervous System" is also a very helpful production. Dr. Ash has dealt with this somewhat difficult subject concisely and adequately enough for the purpose of the book. From these pages a good working knowledge may easily be gained of the main lines on which the human nervous system is constructed, as well as the principles governing its workings. The concluding chapter consists of brief notes on some common diseases of the nervous system. On page 85 there is a misprint of "upper" for "lower." The high reputation of this series is well maintained by the two latest additions to it.

